# A disynaptic basal ganglia connection to the inferior olive: potential for basal ganglia influence on cerebellar learning

**DOI:** 10.3389/fnsys.2023.1176126

**Published:** 2023-05-05

**Authors:** Tom J. H. Ruigrok, Xiaolu Wang, Erika Sabel-Goedknegt, Patrice Coulon, Zhenyu Gao

**Affiliations:** ^1^Department of Neuroscience, Erasmus MC, Rotterdam, Netherlands; ^2^Institute de Neurosciences de la Timone, Centre National de la Recherche Scientifique and Aix-Marseille Université, Marseille, France

**Keywords:** globus pallidus, climbing fibers, cerebellum, mesodiencephalic junction, red nucleus, area parafascicularis prerubralis, entopeduncular nucleus

## Abstract

Recent studies have shown that the cerebellum and the basal ganglia are interconnected at subcortical levels. However, a subcortical basal ganglia connection to the inferior olive (IO), being the source of the olivocerebellar climbing fiber system, is not known. We have used classical tracing with CTb, retrograde transneuronal infection with wildtype rabies virus, conditional tracing with genetically modified rabies virus, and examination of material made available by the Allen Brain Institute, to study potential basal ganglia connections to the inferior olive in rats and mice. We show in both species that parvalbumin-positive, and therefore GABAergic, neurons in the entopeduncular nucleus, representing the rodent equivalent of the internal part of the globus pallidus, innervate a group of cells that surrounds the fasciculus retroflexus and that are collectively known as the area parafascicularis prerubralis. As these neurons supply a direct excitatory input to large parts of the inferior olivary complex, we propose that the entopeduncular nucleus, as a main output station of the basal ganglia, provides an inhibitory influence on olivary excitability. As such, this connection may influence olivary involvement in cerebellar learning and/or could be involved in transmission of reward properties that have recently been established for olivocerebellar signaling.

## 1. Introduction

The basal ganglia and the cerebellum are both important brain structures that are necessary for proper execution of motor behavior (Brooks, 1975; Albin et al., 1989; Thach et al., 1992; Alexander, 1994). Although both structures are also implicated in cognitive, affective, and autonomic functions (Schmahmann, 2010; Koziol et al., 2014; Haber S. N., 2016; Bostan and Strick, 2018) and, as such, cover an incredibly wide range of functions, pathology within either structure usually results in specific disorders that predominantly affect motor functions. The basal ganglia are thought to be involved in the selection and initiation of motor programs based on reinforcement learning, whereas the cerebellum is required for learning, automatizing and coordination of movements. Both structures send a major part of their output to the motor areas of the thalamus and, as a result, will have a prominent effect on the execution of motor programs by their influence on the (pre-) motor cortex. This suggests that both structures are not likely to work independent from one another (Middleton and Strick, 2000). Yet, our understanding how these structures are interacting in order to result in flawless functioning can be considered incomplete at best (Caligiore et al., 2017). Although it is clear that both the basal ganglia and cerebellum receive a major input from the cerebral cortex by way of the corticostriatal and corticopontocerebellar connections, respectively, overlap of reciprocal connections of the basal ganglia output and the cerebellar output to the classical motor part of the thalamus seems to be rather limited (Ilinsky and Kultas-Ilinsky, 1984; Sakai et al., 1996; Kuramoto et al., 2011). Interaction of the output of both systems, therefore, has been considered to take place at the level of the (pre-)motor cortex (Kuramoto et al., 2009; Helmich, 2018).

Yet, it has become clear that at several levels there are also subcortical interactions between the basal ganglia and the cerebellum. Strick and coworkers showed that the cerebellar output, by way of a central thalamic hub, can influence the input stage of the basal ganglia (Hoshi et al., 2005; Bostan and Strick, 2018). More recently, it was shown that this connection might be instrumental in inducing typical basal ganglia syndromes such as dystonia (Calderon et al., 2011; Fremont et al., 2014; Shakkottai et al., 2017). Using tractography, even direct connections between the cerebellum and basal ganglia nuclei have been suggested in human (Milardi et al., 2016, 2019). In addition, a reciprocal connection from the basal ganglia to the cerebellum has been described as the subthalamic nucleus was found to maintain disynaptic connections to the cerebellar cortex, likely with the pontine nuclei as intermediary (Bostan et al., 2010; Pelzer et al., 2013; Milardi et al., 2016; Bostan and Strick, 2018; Ramakrishnan et al., 2022), however, see Jwair et al. for an alternative view (Jwair et al., 2017).

These observations raise the question if the basal ganglia, apart from affecting mossy fibers sources to the cerebellum also could have a subcortical effect on the excitability of the inferior olive (IO), being the source of the cerebellar climbing fiber system. This question seems to be specifically relevant when considering the general notion that activity of the climbing fibers is vital for cerebellar learning (Freeman, 2014; Lisberger, 2021). As the output of the basal ganglia is implicated in action selection based on reinforcement learning (Klaus et al., 2019), it would seem attractive to couple positive actions to improved learning and vice versa. In line with this notion climbing fibers have been shown to signal reward expectation and omission (Kostadinov et al., 2019).

In order to investigate a potential basal ganglia influence on the climbing fiber system, we have analyzed disynaptic afferent pathways to the IO by injecting wildtype rabies virus (wt-RABV) into the IO of the rat. The observed transneuronal labeling of neurons in the entopeduncular nucleus was subsequently verified with single-step tracers in rats and mice and with information available from the Allen Brain Institute. Together, these results show that one of the main basal ganglia output systems has a potentially forceful disynaptic connection to the IO that is mostly mediated by neurons located at the mesodiencephalic junction.

## 2. Materials and methods

### 2.1. Animals

All experiments were carried out on male Wistar rats (*n* = 16, weight 200–250 gr) and both male and female C57BL/6J mice (*n* = 10). The mice used from the Allen Brain dataset were already processed. Animals were 10–20 weeks old and were housed individually in a 12-h light-dark cycle with food and water *ad libitum*. Rat injections with wt-RABV (*n* = 10) were carried out in the BSL2 laboratory of the INT in Marseille, but after perfusion were further processed at the Erasmus MC in Rotterdam. Part of this material was also used in another project (Ruigrok et al., 2016). All other tracing experiments were also performed in Rotterdam. All surgical procedures were carried out in accordance with the European guidelines for the care and use of laboratory animals. The rat experiments in Marseille were furthermore executed in accordance with the guidelines of the French Ministry for Agriculture and Fisheries, Division of Animal Rights and have been approved by the ethics committee in Neuroscience at the INT, Marseille (nr. 02 167.01). Experiments performed in Rotterdam were approved by the institutional animal welfare committee of Erasmus MC (15-273-146 and 15-273-147) in accordance with Central Authority for Scientific Procedures on Animals Guidelines.

In addition to our own injections of tracers in rats and mice, use was made of material available from the Allen Mouse Brain data set^1^ that were accessible before January 2023. At the Mouse Brain Connectivity page,^2^ data from many injections with the anterogradely transported viral vectors AAV-GFP and AAV-FLEX(DIO)-GFP in wild-type and transgenic mice, respectively, can be found and evaluated. Three cases, all using different transgenic lines, were available with injections restricted to the entopeduncular nucleus (EP), which is referred to as internal part of the globus pallidus in the Allen Brain Connectivity atlas. We have examined Experiment 305024724-GPi in the retinol binding protein 4 (Rbp4-Cre_KL100) transgenic mouse^3^ and Experiment 539498984-GPi in the somatostatin (Sst-IRES-Cre) mouse line.^4^ The Rbp4-Cre mouse line is mentioned to be enriched in the deeper layers of the cerebral cortex and in the dentate gyrus. However, from the transgenic characterization data, it can be observed that many neurons within the EP also are Rbp4-Cre-positive. The transgenic characterization of the Sst-IRES-Cre mouse also shows positive neurons scattered within and around the EP. By using AAV-FLEX(DIO)-GFP viral tracer in these transgenic mouse lines only Rbp4- and Sst- positive neurons are expected to produce anterograde labeling. Further details of used procedures are available at the website (see text footnote 2).

### 2.2. Wt-RABV injections in the inferior olive of the rat (*n* = 10)

In order to visualize 2nd-order afferents of the IO, we have used the “French” subtype of CVS-11 strain of RABV (wt-RABV: titrating 4 × 10^7^ plaque-forming units/ml). This wt-RABV strain is known to be transported transneuronally in a retrograde direction and in a time-dependent fashion (Ugolini, 1995, 2020; Ruigrok et al., 2008; Aoki et al., 2019). No neighboring neurons are infected unless they maintain synaptic contacts to the already infected cells. Indeed, infected neurons remain morphologically intact for at least several days, making risk for false-positive infection of non-related neurons or fibers minimal (Ugolini, 1995; Ruigrok et al., 2008; Aoki et al., 2019). For rats, and using injections in either cerebellar or cerebral cortex, it was established that only 1st-order retrograde infection can be observed 30 h post-injection. Subsequent transneuronal steps to result in 2nd- and 3rd-order labeling each take approximately 20 h (Suzuki et al., 2012; Ruigrok et al., 2016; Aoki et al., 2019).

Surgical procedures have been described before (Suzuki et al., 2012; Ruigrok et al., 2016; Aoki et al., 2019). Briefly, rats were anesthetized with a ketamine/xylazine mixture. Animals were placed in a Kopf stereotaxic frame and the skin overlying the occipital bone and neck was incised, neck muscles were separated in the midline and the foramen magnum was somewhat enlarged in dorsal direction using a rongeur. In this way, the obex became visible and served as a landmark for olivary injections (Ruigrok and Voogd, 2000).

The wt-RABV stock was kept at −80°C until use. The injectate consisted of a mixture of 4 parts wt-RABV and 1 part cholera toxin β-subunit [CTb, low salt; List Biological Laboratories, 1% w/v in 0.2 M phosphate buffer (PB) at pH 7.4]. CTb was added to accurately evaluate the injection site and to be able to discriminate between non-transneuronal (CTb) and non-transneuronal and transneuronal infection by the wt-RABV (Ruigrok et al., 2008; Suzuki et al., 2012). Injection of approximately 150 nl was made with a 1 μl Hamilton needle attached to a microsyringe pump (Ruigrok et al., 2016). The IO was reached at a −45° angle with the vertical axis, with the needle entering at 0.6–0.8 mm lateral to obex at a depth of 2.7–2.8 mm. After injection the neck muscles were sutured in layers, the skin was clamped and the animal was allowed to recover.

After survival times of 30 (*n* = 1), 48–50 (*n* = 6), or 66–70 (*n* = 3) h, during which the rats did not behave differently from other experimental animals with tracer injections, animals were deeply anesthetized with an overdose pentobarbital (100 mg/kg i.p.: Nembutal, CEVA, Santé Animale, Libourne, France), the thorax was opened and the animal was transcardially perfused with an initial rinse of 0.9% saline (200 ml) followed by 300–400 ml of freshly prepared 4% paraformaldehyde in phosphate buffer with saline (0.9%: PBS). Brain and spinal cord were subsequently removed, post-fixed for several days to completely inactivate the virus (Kelly and Strick, 2000), rinsed overnight in PB with 10% sucrose and embedded in gelatin (11% in 30% sucrose), which was hardened in 10% formalin with 30% sucrose for 3 h. After an overnight rinse in PB with 30% sucrose, the gelatin blocks were cut coronally at 40 μm with a freezing microtome (Leica SM2000R). Sections were collected serially in eight numbered glass vials.

### 2.3. CTb injections in the APP of the rat (*n* = 6)

Surgical procedures for injecting tracer in the area parafascicularis prerubralis (APP) in rats have been described by Ruigrok and Teune (2014). After anesthetizing and mounting the animal as described above, the APP area was approached through a small hole drilled in the parietal bone directly overlying the APP (approximately 5.0 mm rostral to the interaural line, laterality 0.8–1.0 mm). CTb (1% w/v in 0.1 M PB) in the APP area was delivered iontophoretically by means of a 4 μA positive current for 10–15 min. (7 s on/off cycle). After clamping the skin, animals were allowed to recover and survived for 4 or 5 days. Termination of the experiment and extraction and sectioning of the brain was as described above.

### 2.4. CTb injections in the APP of the mouse (*n* = 6)

Surgical procedures for injecting CTb in the APP region of the mouse have been described by Wang et al. (2022). Briefly, mice were anesthetized by inhalation of 5% isoflurane for induction and 2.5% for maintenance. Animal head was positioned in a holder such that bregma and lambda were at the same horizontal level. A small vertical incision was applied on the skin to expose the parietal bones and a small craniotomy (diameter: ∼0.7 mm) was made. Based on stereotaxic coordinates and using a glass capillary (tip diameter: ∼8 μm) approximately 30 nl CTb solution (1%, Sigma-Aldrich, C9903) was slowly injected. Animals survived for 2 days after which they were deeply anesthetized by intraperitoneal injection of pentobarbital and perfused transcardially with saline and paraformaldehyde. Brains were post-fixed for 2 h, embedded in gelatin and sectioned coronally with a freezing microtome at 50 μm.

### 2.5. Conditional monosynaptic RABV tracing in mice (*n* = 4)

Surgical procedures for the conditional monosynaptic tracing with genetically modified RABV (Figure 5) have been described by Wang et al. (2022). Initially, helper virus (AAV8-CAG-FLEX-TCB: encoding TVA gene and AAV8-CAG-FLEX-oG, Salk Vector Core, mixed 1:1 in volume) were simultaneously delivered in the APP and AAVretro-hSyn-Cre-eBFP (Addgene) was injected into the IO. After allowing for transport and expression for 4 weeks the modified RABV (RABV-CMV-EnvA–ΔG-eGFP, Charite Vector Core) was injected in the APP. Mice were sacrificed 1 week after the RABV injection as described above.

### 2.6. Histological procedures

As all brain sections were serially collected in 4 (mice) or 8 (rats) vials, selected vials, each containing a complete set of equidistant serial sections, could be chosen for a specific histological procedure.

For CTb immunohistochemistry, sections were first rinsed with phosphate-buffered saline containing 0.9% NaCl, and incubated in 3% hydrogen peroxidase (H2O2) in PBS for 20 min to quell endogenous peroxidase activity. Sections were subsequently incubated in goat anti-CTb subunit primary antibody (1:15,000, List labs, 703) diluted in PBS+, that is, PBS containing 2% normal horse serum and 0.5% Triton X-100, followed by incubation with biotinylated horse anti-goat secondary antibody (1:2,000, Vector, BA-9500) for 2 h at room temperature. Slices were incubated with ABC complex (volume ratio 1:1, Vector, AK5200) for 1.5 h at room temperature. Next, labeling was visualized with 3,3′-diaminobenzidine tetrahydrochloride (DAB) solution (0.025% DAB and 0.005% H2O2 in 0.05 M PB) for 20 min at room temperature, generating a brown insoluble reaction product in CTb-labeled structures, and slices were mounted sequentially, Nissl-counterstained with thionine and cover-slipped with Permount.

For wt-RABV immunohistochemistry, sections were rinsed, pretreated with peroxidase and incubated overnight at room temperature in an anti-rabies phosphoprotein mouse monoclonal antibody (Raux et al., 1997; Suzuki et al., 2012) diluted at 1:5000 in PBS+. Subsequently, the sections were incubated in secondary rabbit anti-mouse horseradish peroxidase (P260 Dako, 1:200 in PBS+), followed by DAB visualization.

Some vials, selected for dual fluorescent immunolabeling of wt-RABV and CTb were incubated for 48–72 h (4°C) with a mixture of the monoclonal anti-RABV and goat anti-CTb in PBS+. After rinsing sections were incubated for 2 h with the secondary antibodies donkey anti-goat-Cy3, 1:200 and donkey anti-mouse-FITC, 1:200 in PBS+ (Jackson ImmunoResearch Europe, Inc.). Sections for fluorescent microscopy (FM) were also mounted serially from chromic alum, coverslipped with Vectashield HardSet Mounting Medium (H-400, Vector Laboratories) and stored in the dark in dust free boxes at 4°C.

For somatostatin (Sst), parvalbumin (Parv) and vglut2 triple fluorescent immunolabeling, sections were incubated in rabbit anti-parvalbumin (1:10000, Swant, 235) with either goat anti-somatostatin (Sst, 1:1000, Santa Cruz, sc-7819) or anti-vesicular glutamate transporter 2 (guinea pig anti-vglut2 primary antibody, 1:1000, Sigma, AB2251-I), followed by Cy™ 5 donkey anti-rabbit (1:400, Jackson, 711-175-152) and either Cy™3 donkey anti-goat (1:400, Jackson, 705-165-147) or Alexa fluor donkey anti-guinea pig (1:400, Jackson, 706-545-148) secondary antibodies.

### 2.7. Analysis

3,3′-diaminobenzidine tetrahydrochloride-incubated material was examined and photographed with a Leica DMR microscope (Nussloch, Germany) equipped with a digital camera (Leica DFC-450) or with a Nanozoomer (2.0-RS, Hamamatsu). For plotting and 3D- reconstructions a motorized Olympus BH microscope and Neurolucida™ software (Microbrightfield) was used. Fluorescent sections were assessed and photographed with Axio Imager 2 equipped with slide scanner (Zeiss). Fluorescent sections were examined and optimized using Zen 2 (blue edition: Zeiss) software and the open-source image processing package Fiji. Photo panels were constructed with Illustrator™ 2022 (Adobe Creative Cloud). Evaluation of the level of the sections (with respect to bregma) and delineation of structures was aided by the atlases of the mouse and rat brain (Paxinos and Franklin, 2004; Paxinos and Watson, 2007).

Flattened maps of CTb and wt-RABV labeled neurons (Figures 2A2, B2) are based on sequential series of plots of one of eight sections (i.e., every 320 μm). Subsequently, for every plot, an 80-μm wide rectangle overlay was used to count the number of labeled neurons within every mediolateral bin. Thus, every bin resulted in the number of labeled structures in a dorsoventral column of 80 μm wide and 40 μm in anteroposterior direction through the brainstem. The obtained numbers served as a measure of the density of labeled neurons. Using standard MATLAB™ (MathWorks) routines and interpolation (Ruigrok, 2003; Pijpers and Ruigrok, 2006), the data were visualized as color-coded density plots in which the contours of several structures were also indicated by interpolation.

## 3. Results

### 3.1. CTb/RABV injections in the inferior olive of the rat

In order to study potential pathways from the basal ganglia to the IO, the “French” strain of CVS11 (wt-RABV) was mixed with CTb and pressure-injected into the right inferior olivary complex of male Wistar rats (*n* = 11). Survival times were chosen to result in either 1st-order (30 h, *n* = 1), 2nd-order (48–50 h, *n* = 6) or 3rd-order (66–70 h, *n* = 3) transneuronal retrograde infection of neurons with wt-RABV. Some of these experiments were used in another project which studied the connections between the cerebellar nuclei and the IO (Ruigrok et al., 2016).

**FIGURE 1:**
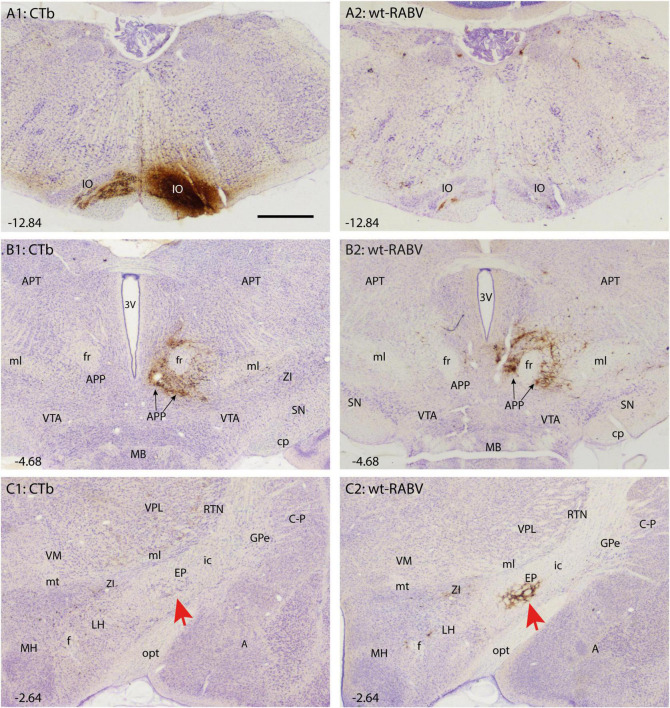
Comparison of CTb and wt-RABV labeling 48 h after injection of the right IO of the rat. **(A1,A2)** Show the injection site using DAB immunostaining for CTb and RABV, respectively. Note that the injection site could only be evaluated by CTb staining. As expected only very sparse wt-RABV labeling was present within the IO (Aoki et al., 2019). **(B1,B2)** Depict the mesodiencephalic junction where many CTb-labeled as well as wt-RABV-infected neurons are found surrounding the ipsilateral fr together forming the APP. **(C1,C2)** In the diencephalon, no CTb labeling is observed in the EP (red arrow) or anywhere, whereas ample staining of RABV-infected neurons is observed in the ipsilateral EP (red arrow). 3V, third ventricle; A, amygdala; APP, area parafascicularis prerubralis; APT, anterior pretectal nucleus; C-P, caudate-putamen; cp, cerebral peduncle; EP, entopeduncular nucleus; f, fornix; fr, fasciculus retroflexus; GPe, globus pallidus external part; ic, internal capsule; IO, inferior olive; LH, lateral hypothalamus; MB, mammillary body; MH, medial hypothalamus; ml, medial lemniscus; mt, mammillothalamic tract; opt, optic tract; RT, reticular thalamic nucleus; SN, substantia nigra; VM, ventromedial thalamic nucleus; VPL, ventral posterolateral thalamic nucleus; VTA, ventral tegmental area; ZI, zona incerta. Approximate position of sections with the respect to bregma is indicated in lower left hand corner (Paxinos and Watson, 2007). Scale bar equals 1 mm.

CTb-immunohistochemistry enabled assessment of the IO injection site as well as of the direct afferent (by retrograde transport) and efferent (by anterograde transport) connections of the neurons within the injection site. The location and size of the injection site was judged from the dense part of CTb injection site as visualized by DAB-immunohistochemistry as wt-RABV immunohistochemistry was inadequate to delineate the injection site (Figures 1A1, A2; Prevosto et al., 2009; Suzuki et al., 2012). In all cases the injection was clearly centered on the inferior olivary complex, with only marginal incorporation of the surrounding reticular formation or the pyramidal tract. This was further verified by massive anterograde CTb labeling of climbing fibers in the contralateral cerebellar cortex with virtually no labeling of mossy fiber rosettes (Supplementary Figure 1).

**FIGURE 2:**
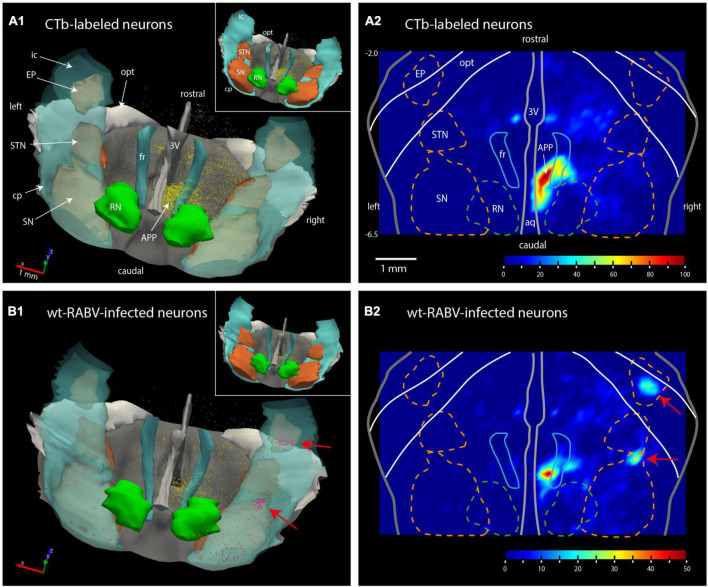
Plots of the rat mesodiencephalic region showing wt-RABV, but not CTb, labeling in EP and rostral SN. **(A1,B1)** Show 3D-renderings of the mesodiencephalic region from a dorsolateral and caudal viewpoint based on a series of 1 out 4 plotted sections. Yellow dots represent single labeled neurons, red dots represent labeled neurons located in the SN and EP. Nuclear boundaries of the SN and STN are made solid in the insets. **(A2,B2)** Show the same area in a dorsoventrally flattened surface rendering. Contours of several nuclei as they appear in such a flattened rendering are shown. Density of labeling is indicated by color-coding. Note that both for CTb and for RABV high density labeling is apparent around the right fr. RABV labeling is furthermore present in the rostral tip of the SN and within the confines of the EP. aq, aqueduct of Sylvius; RN, red nucleus; STN, subthalamic nucleus. For other abbreviations: see legend to Figure 1. Scale bar equals 1 mm; –6.5, –2.0 approximate anterior-posterior levels with respect to bregma.

Retrogradely CTb-labeled cell bodies of well-known afferents of the IO (Ruigrok et al., 2015) were observed in all animals within the spinal cord, superior colliculus, dorsal column and trigeminal nuclei, pretectal nuclei and, very prominently, at the mesodiencephalic junction surrounding the central aspect of the fasciculus retroflexus (fr: Figure 1B1). Although the latter area covers several separately labeled nuclei, it conjunctively has been described as the nucleus parafascicularis prerubralis (Carlton et al., 1982). However, as this region is difficult to separate from surrounding areas purely based on cytological grounds and therefore does not appear as a well-delineated nucleus, we will refer to this region as the APP (Ruigrok et al., 2015). Importantly, in all animals no CTb labeling was observed anywhere within the basal ganglia (Figure 1C1).

Wildtype rabies virus infection after a 30 h survival time was limited to the same areas where CTb-labeled cell bodies could be recovered, without any infection of neurons in the basal ganglia. Hence, at this time interval only 1st-order infection had occurred without any evidence for transneuronal infection. When the survival time was increased to 48–50 h to allow for a single transneuronal retrograde step, all 1st-order areas were also labeled, be it with a somewhat higher density of both the number of labeled neurons and intensity of labeling (Figure 1B2). Indeed, fluorescent double labeling showed that many CTb-labeled neurons in the APP also were infected with wt-RABV (Supplementary Figure 2). However, and unexpectedly, at this time also prominent RABV infection was observed within the entopeduncular nucleus (EP: Figure 1C2), which is the rodent equivalent of the internal part of the globus pallidus (Haber and Nauta, 1983; Bolam et al., 2000). Wt-RABV-infected cells were also encountered within the reticular part of the substantia nigra, especially rostrally. Figure 2 shows a 3D-reconstruction and a color-coded flattened representation of CTb and wt-RABV labeling within the mesodiencephalic area in case 1100 with a survival time of 48 h. 2D-plots of this case are shown in Supplementary Figures 3A, B. In this and other cases with a 48/50-h survival time, no wt-RABV infection was observed in any other part of the basal ganglia (i.e., external part of the globus pallidus, subthalamic nucleus, and caudate-putamen). All these areas became prominently labeled with infected neurons in three animals where the survival time was lengthened to 66–70 h to allow for an extra transneuronal transfer of wt-RABV (Supplementary Figure 4). It was concluded that the EP neurons infected with wt-RABV after 48–50 h represent 2nd-order labeling resulting from the IO injection.

### 3.2. CTb injections in the mesodiencephalic junction of the rat and mouse

**FIGURE 3:**
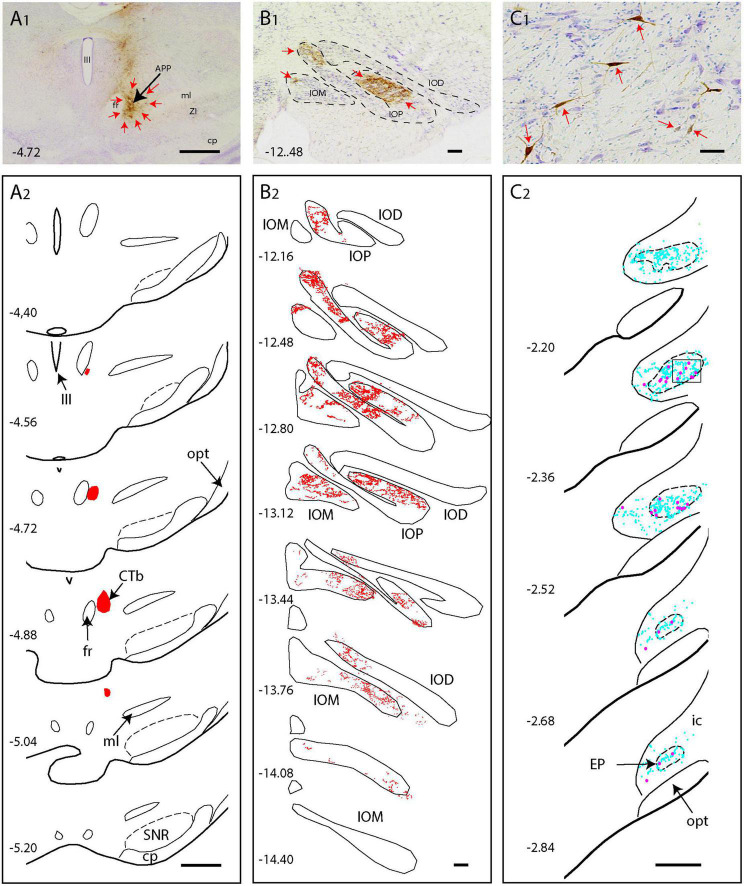
CTb injection in the APP of the rat results in anterograde labeling in the IO and retrograde labeling in the EP **(A1,A2)** Photo and series of diagrams indicating the extent of the CTb injection [red arrows in panel **(A1)**], which is confined to the APP region. **(B1,B2)** Photograph and series of plots of anterograde terminal labeling of CTb labeled fibers in the inferior olive. Note dense labeling in IOP and IOM (red arrows). **(C1,C2)** Microphotograph and series of plots showing retrograde labeled neurons (red arrows and magenta dots) in the EP. Cyan dots represent non-labeled neurons within the EP area. Scale bars represent 100 μm in **(A1,A2)** 1 mm in panels **(B1,B2)**, and panels **(C1,C2)**. Numbers refer to anteroposterior level with respect to bregma. IOD, dorsal accessory nucleus of the inferior olive (IO); IOM, medial accessory nucleus of IO; IOP, principal nucleus of IO, for other abbreviations: see legend to Figure 1.

Examination of CTb labeling of all cases did not show any retrogradely labeled neurons in the classic output nuclei of the EP, i.e., the motor regions of the thalamus and the habenula (Rajakumar et al., 1994; Wallace et al., 2017). This indicates that the 2nd order wt-RABV labeling in the EP could not occur as a result of virus transfer from these classic EP output structures but must be found in the areas containing CTb retrograde labeling. As the area surrounding the APP was the most densely labeled area, we proposed that this region was the most likely candidate to act as an intermediate for the wt-RABV to reach the EP. This possibility was verified in rat by injecting CTb into the APP (*n* = 6). Indeed, when the injectate encompassed the APP, dense anterograde labeling was noted in large areas of the inferior olivary complex, but also retrogradely labeled neurons were observed in the ipsilateral EP (Figure 3). These injections were repeated in the mouse (*n* = 6), with the same results (Supplementary Figures 5A, B). In one mouse, where the injection was centered on the PAG area just medial to the APP, no IO anterograde labeling, nor EP retrograde labeling was observed (Supplementary Figure 5C), indicating that the PAG was not a likely intermediary between EP and IO. These experiments indeed suggest that the APP region receives EP input and project to the IO.

### 3.3. EP projects to APP in the mouse (Allen Brain Atlas)

**FIGURE 4:**
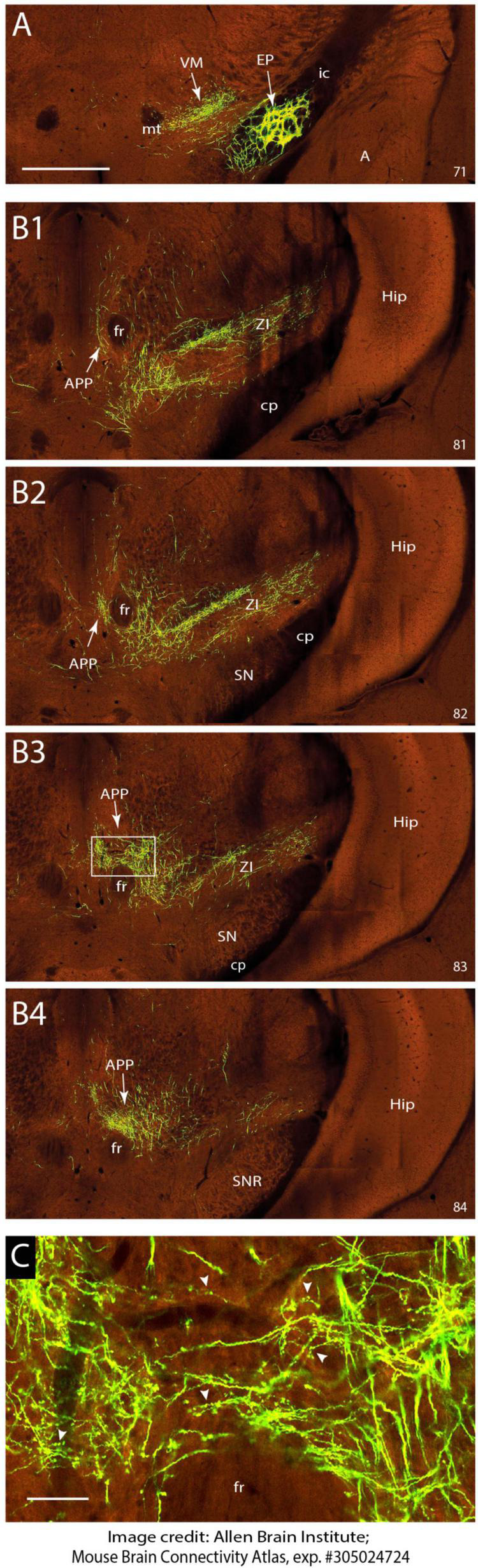
EP sends terminal arborizations to the APP in the mouse. **(A)** Injection site of anterograde transported AAV in the EP. Anterograde labeling in the VM area are fibers and terminal arborizations. **(B1–B4)** Serial photographs of the mesodiencephalic region showing from rostral **(B1)** to caudal **(B2)** the APP area surrounding the fr. Note the prominent labeling around the fr. **(C)** Magnification of the boxed area in B3 showing the varicose terminal arborizations of labeled fibers in the APP. Images are from the Mouse Brain Connectivity Atlas, exp. #305024724, (see https://connectivity.brain-map.org/projection/experiment/305024724; Allen-Institute-For-Brain-Science, 2004). Numbers in lower right-hand corner refer to image number. See Section “Materials and methods” for further details. For abbreviations: see legend to Figure 1. Scale bar equals 1 mm for panel **(A)** and **(B1–B4)** and 100 μm for panel **(C)**.

As retrograde labeling of EP neurons could be due to inadvertent uptake of EP fibers that pass the APP region to the pedunculopontine tegmental nucleus (Semba and Fibiger, 1992; Steininger et al., 1992) and retrograde labeling of somata does not provide information on the density of the axonal projections, we sought for a way to examine anterograde tracing from the EP. This was enabled by scanning the Mouse Brain Connectivity atlas of the Allen Brain Atlas (Allen-Institute-For-Brain-Science, 2004). Here, three injections in different mouse genotypes with an anterogradely transported adeno-associated virus (AAV) that incorporated the EP can be accessed (note that the EP is labeled as internal segment of the globus pallidus in Allen Brain Atlas). As only 1% of the injection site of case 305024724-GPi (Rbp4-Cre_KL100 mouse line see text footnote 3) was reported to cover the zona incerta while 97% covers the EP, this case is not likely to be hampered by a considerably amount of false positive labeling. Figure 4 shows the injection site and serial photographs of the mesodiencephalic region. From these, it can be seen that many fibers emanating from the EP as ansa lenticularis, pass through field H of Forel. Here, while most fibers turn dorsolateral toward the ventromedial and ventrolateral thalamus, many fibers dissociate themselves from the main bundle and follow a dorsocaudal route to distribute many terminal arborizations to the APP (Figures 4B3, B4, C). Hence, we conclude that EP can be considered as a major source of afferent information for the APP.

**FIGURE 5:**
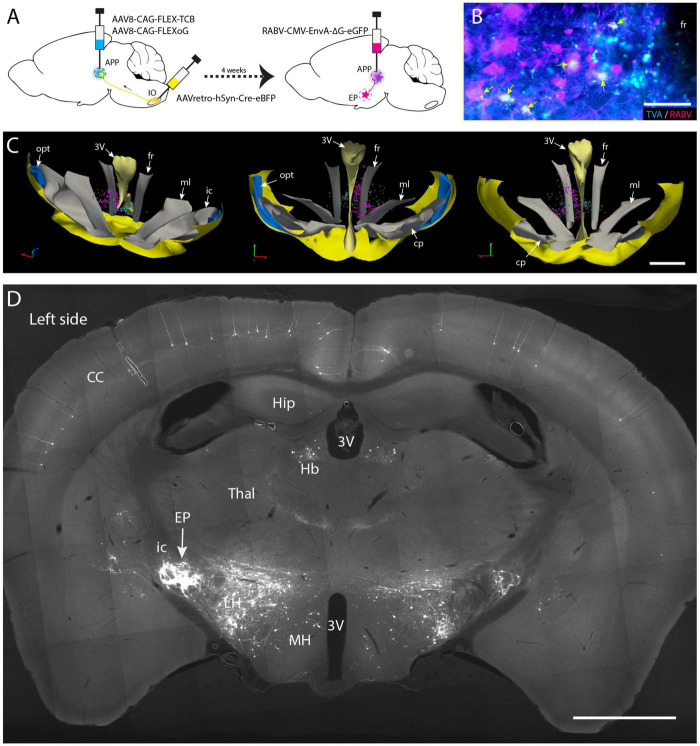
Conditional monosynaptic RABV tracing revealing EP input on IO projecting APP neurons in the mouse. **(A)** Schematic of the transneuronal viral tracing strategy. Helper virus conditionally expressing TVA and oG are injected into the left APP. Neurons will only express TVA and oG when they have incorporated retrogradely transported Cre from the ipsilateral IO. After a 4-week incubation period, these cells can become starter cells for monosynaptic tracing after APP injection with genetically modified RABV expressing ENVA rather than G and an additional week of incubation. In starter cells recombination of modified RABV with oG enables a single transneuronal retrograde step, resulting in the labeling of afferents of the starter cells. **(B)** Double labeling of starter cells in the fr (arrows) of a representative mouse. **(C)** 3D-reconstruction of starter cells (magenta) in the mesodiencephalic junction of the same mouse shown in panel **(B)** (based on a 1 out of 4 series of plots of sequential sections). Starter cells are only found in the APP region surrounding the fr. Cyan markers represent TVA-expressing neurons but are negative for RABV. From left to right the 3D-reconstruction is seen from a dorsolateral caudal, a rostral, and a caudal viewpoint, respectively. **(D)** Photomicrograph of a coronal section of resulting RABV labeling at the level of the EP. Note dense labeling of the EP ipsilateral to the injections (left side). Some labeling is also present in the contralateral EP (right side). Additional labeling can be observed in the LH, Hb and of layer V pyramidal cells in the CC. For abbreviations, see legend to Figure 1. Scale bars represent 100 μm in panel **(B)** and 1 mm in panels **(C,D)**.

### 3.4. EP projects to IO projecting neurons in APP

Most, if not all, of the APP neurons that project to the IO are excitatory (De Zeeuw et al., 1990; De Zeeuw and Ruigrok, 1994; Ruigrok and Voogd, 1995), yet other cell types also may be present within the APP. Furthermore, our wt-RABV experiments were conducted in rat, while confirmation of an EP-APP connection was obtained in mice. Therefore, in order to further verify that also in mice EP neurons project to APP neurons that provide input to the IO, a conditional gene-modified transneuronal rabies (RABV) tracing strategy was used (Callaway and Luo, 2015; Wang et al., 2022). First, a Cre-dependent helper virus (AAV8-CAG-FLEX-TCB, encoding TVA gene) together with a virus that expresses the rabies (RABV) membrane-bound glycoprotein (G: AAV8-CAG-FLEX-oG) were injected into the APP region (Figure 5A). Simultaneously, AAVretro-hSyn-Cre-eBFP was injected into the ipsilateral IO complex. This allows APP neurons that project to the IO to express both rabies glycoprotein (oG) and the avian receptor TVA. Next, after 4 weeks incubation, glycoprotein-deleted rabies RABV, pseudotyped with the avian glycoprotein EnvA (RABV-CMV-EnvA-ΔG-eGFP) was injected in the APP (*n* = 4) and allowed to incubate for another week. With this approach only neurons expressing TVA and thus projecting to the IO could be infected with the ΔG-RABV. In this way, a group of starter cells was labeled from which, after reconstitution with oG, a single retrograde transneuronal step was permitted. Starter cells, double labeled with RABV and TVA, indeed were most prominently found around the fr (Figures 5B, C; Supplementary Figure 6). Transsynaptic RABV labeling was observed in many locations such as the zona incerta, cerebral cortex and lateral hypothalamus (Figure 5D), as well as in the cerebellar nuclei (Wang et al., 2022). Importantly, many RABV-labeled neurons were also encountered in the EP (Figure 5D). This confirms our hypothesis that neurons in the APP form a major intermediary in a disynaptic EP-APP-IO connection.

### 3.5. EP projections to APP are parvalbumin-positive and somatostatin-negative

**FIGURE 6:**
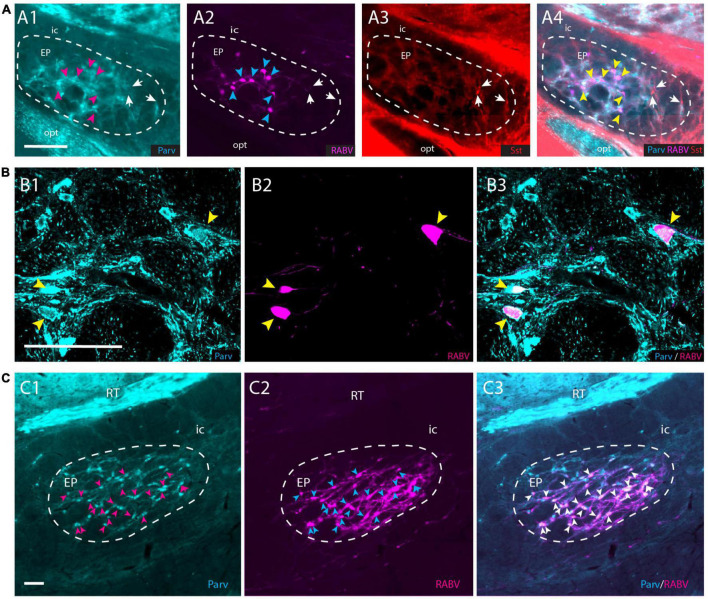
RABV-infected neurons in the EP are Parv+ in both rats and mice. **(A1–A4)** RABV-labeled neurons in the EP **(A2**) labeled after the tracing strategy in mice as described in Figure 5A, are Parv-positive and Sst-negative. Magenta arrowheads indicate the position of RABV+ neurons in panel **(A1)**; cyan arrowheads indicate RABV+ cells in panel **(A2)**; yellow arrowheads refer to the position of RABV+ cells in panel **(A4)**; white arrows indicate Sst-positive neurons in panels **(A3,A4)** and location of those cells in panels **(A1,A2)**. **(B1–B3)** Confocal image showing RABV and Parv colocalization in EP. Yellow arrowheads indicate double-labeled neurons. **(C1–C3)** Similarly, in the rat EP, using wt-RABV injected in the IO and a survival time of 50 h all RABV-infected neurons (cyan) arrowheads in panel **(C2**), also contain Parv (indicated by magenta arrowheads in panel **(C1)** and white arrowheads in panel **(C3)**. Parv, parvalbumin; Sst, somatostatin; for other abbreviations see legend to Figure 1. Scale bars equal 100 μm.

Although the EP is well-known for its GABAergic projection to motor-related areas in the thalamus, it is well-established that other types of projection cells also can be found within the confines of the EP (Vincent and Brown, 1986; Wallace et al., 2017; Miyamoto and Fukuda, 2021). Parvalbumin-positive (Parv+) neurons are GABAergic and project to the ventrolateral thalamus, whereas somatostatin-positive (Sst+) neurons contain GABA and/or glutamate and target the lateral habenula (Vincent and Brown, 1986; Rajakumar et al., 1994; Wallace et al., 2017). In order to determine which group of EP neurons target the APP-IO neurons, we have used a triple-labeling fluorescent immunohistochemical procedure of the conditional RABV tracing experiments described in the former section, to determine if the RABV-positive neurons in the mouse EP were Parv+ or Sst+ or Vglut2+. Confocal microscopy confirmed that all RABV-infected neurons in the EP were Parv+ (Figures 6A, B). No double labeling of RABV with either Sst or VGlut2 was observed (Supplementary Figures 7A, B). In addition, also in the rat material, double-immunolabeling, 48 h after wt-RABV injection in the IO, indicated that wt-RABV-transfected EP neurons were all Parv+ (Figure 6C). Finally, we noted that case 539498984-GPi available from the Allen Brain Atlas (see text footnote 3; Allen-Institute-For-Brain-Science, 2004), with an AAV injection in the EP of a Sst-IRE-Cre mouse-line, thus limiting anterograde transport to Sst-containing neurons the EP, did not show any labeling within the APP (Supplementary Figure 8). Rather, labeled fibers exit the GPi by way of the lentiform fasciculus, terminating mostly in the parafascicular thalamic nucleus or, by way of the thalamic stria medullaris, reach the habenula. Together with our double- and triple-labeling in rat and mouse experiments, respectively, this makes it highly unlikely that Sst- and/or Vglut2-positive neurons in EP project to the APP. We conclude that the EP input to APP-IO projecting neurons originates from Parv+, and therefore GABAergic, neurons that, most likely, also project to the motor thalamus (Rajakumar et al., 1994; Wallace et al., 2017).

## 4. Discussion

We have conducted a number of tracing experiments in rats and mice to investigate if the basal ganglia has a subcortical connection to the cerebellum by way of the climbing fiber system.

### 4.1. EP connects disynaptically to IO by way of the APP

Our results obtained in the rat show that many neurons in the EP, which constitutes an important output source of the basal ganglia, become infected 48 h, but not yet 30 h, after injection of wt-RABV in the IO. Wt-RABV specifically enters afferent terminals in the injection area resulting in retrograde infection of 1st-order neurons after about 30 h. Transneuronal steps to subsequent 2nd and higher order neurons are time-dependent and, depending on species and viral strain, typically take 20–24 h per step. Post-injection survival times, therefore, need to be critically evaluated in order to monitor the progression of the virus throughout the brain (Ugolini, 1995, 2020; Suzuki et al., 2012; Aoki et al., 2019). Assessment of the progression of infection to the EP, therefore, is perfectly in line with 2nd-order transneuronal labeling from the IO, suggesting a disynaptic connection from the EP to the IO.

Our results further present evidence that the APP, a large group of neurons centered around the fr (Carlton et al., 1982), serves as an intermediary for this EP-IO connection. CTb injections in the APP in both rats and mice result in anterograde terminal labeling within the IO and, simultaneously, in retrograde neuronal labeling in the EP. Also, analysis of mouse anterograde viral injections in the EP, available from the Allen Brain Atlas, shows robust terminal arborizations in the APP. Finally, we provide evidence for the inhibitory nature of the EP-APP connection as wt-RABV infected neurons in the rat EP were all Parv+ and therefore likely to be GABAergic (Rajakumar et al., 1994). This was collaborated in our mice experiments with conditional transport of modified RABV that additionally showed that RABV-infected neurons in the EP were Sst- and Vglut2-negative.

Together, these data present evidence for a strong GABAergic input from the EP to a group of neurons that provide an excitatory connection to a large part of the IO. As our experiments allow to reach a similar conclusion on the prominence of a disynaptic nature from the EP to the IO in both mice and rats, we suggest that this may very well be extrapolated to other mammalian orders. The GABAergic nature of the EP-APP connection would indicate that the basal ganglia output could inhibit an excitatory drive to the IO.

### 4.2. Position of the APP

In cat and in primates, several nuclei at the mesodiencephalic junction provide a major projection to the IO, where especially the principal olive and rostral part of the medial accessory olive have been described as major targets (Carlton et al., 1982; Swenson and Castro, 1983a,b; Onodera, 1984; Onodera and Hicks, 2009). This projection seems to culminate in human, where the prominent parvicellular red nucleus supplies a major projection to the IO, while its magnocellular part supplying the crossed rubrobulbar and -spinal tracts has dwindled (Nathan and Smith, 1982). Conspicuously, in rats both magno- and parvicellular parts of the red nucleus do not provide a projection to the olive (Rutherford et al., 1984). As the human fr passes the rostromedial part of the parvicellular red nucleus (Paxinos et al., 2012), it has been suggested that the APP is similar in position as well as in function as the human parvicellular red nucleus (Ruigrok and Cella, 1995).

In this respect, it is interesting to relate our results with the alleged, but somewhat elusive, pallidorubral projection that has been described in older literature (Wilson, 1914; Vogt and Vogt, 1920; Johnson and Clemente, 1959), but that was also specifically denied by many other authors of that era (Von Monakow, 1909; Verhaart, 1938; Papez and Stotler, 1940). Nauta and Mehler, in their seminal review (Nauta and Mehler, 1966), reported that pallidorubral fibers are extremely sparse in monkey. A notion that was not contradicted in a recent commentary to Nauta and Mehler’s paper by Haber S. (2016). Hence, there seems to be general consensus that the descending fibers from the internal segment of the globus pallidus (i.e., the EP) mostly reach the substantia nigra and pedunculopontine reticular nucleus (Haber S., 2016). As far as we know, in this century a pallidorubral connection was only specifically mentioned in a few studies using diffusion tractography in human (Blood et al., 2012; Waugh et al., 2019). Yet, in light of the vast expansion of the parvicellular red nucleus in apes and human (Onodera and Hicks, 2009; Hicks and Onodera, 2012), a similar increase in pallidal input to this area might also be expected.

Despite the sparseness of previously published data on a projection from one of the main output structures of the basal ganglia to a group of neurons that seem to exclusively target the IO (Onodera, 1984), we are confident that this projection has now been clearly established in the present set of experiments carried out in rodents. As such, it may play an important role in cerebellar functioning that may complement other inputs from the cerebellum to the basal ganglia (Bostan et al., 2010; Pelzer et al., 2013; Milardi et al., 2016; Bostan and Strick, 2018; Krzyspiak and Khodakhah, 2022). It furthermore supplements and highlights the subcortical reciprocity of interconnections between cerebellum and basal ganglia (Bostan and Strick, 2018).

### 4.3. Functional implications of an inhibitory EP-APP connection

Apart from the afferents derived from the EP, the APP receives a vast input from the cerebral cortex, but also from the cerebellum (De Zeeuw and Ruigrok, 1994; Wang et al., 2022). Indeed, stimulation of the cerebral cortex as well as of the cerebellar nuclei can activate olivary units (Crill, 1970; Ruigrok and Voogd, 1995; Pardoe et al., 2004; Watson et al., 2009). Presently, not much is known how the APP, or parvicellular red nucleus of primates, integrates signals from cerebrum and cerebellum in order to provide a meaningful excitatory drive to the IO. Yet, as the importance of the IO as an instructor for cerebellar learning seems to be beyond doubt (De Zeeuw, 2021; Lisberger, 2021; De Zeeuw and Romano, 2022), insight in the control of olivary excitation will be of the utmost relevance for understanding cerebellar learning mechanisms.

This is especially true when considering important aspects of the olivary physiology, such as the subthreshold oscillations of the membrane potential, the relatively slow firing frequency of olivary units, and the alleged importance of timing and synchrony of olivary discharges (De Zeeuw and Romano, 2022). We now see that olivary excitation is not just dependent on both a direct GABAergic input from the cerebellar nuclei (De Zeeuw et al., 1989) as well as on an excitatory disynaptic loop from the same cerebellar nuclei via the APP (Ruigrok and Voogd, 1995; Wang et al., 2022), but that the APP throughput can be modulated by an excitatory input from layer V pyramidal cells of the cerebellar cortex (Wang et al., 2022) and by a GABAergic, and therefore presumably inhibitory, input from the EP.

It is tempting to speculate on the function of the EP-APP-IO connection. Although not demonstrated in the present set of experiments, from the sheer number of labeled EP neurons in the rat experiments with wt-RABV, it seems likely that the EP-APP fibers are collaterals from the fibers that reach the motor parts of thalamus (Wallace et al., 2017). This projection is thought to curb or allow actions based on reinforcement learning (Graybiel, 1995). A strong inhibitory input to the motor thalamus is believed to inhibit thalamocortical interactions, thereby interfering with movement execution by the motor cortex. When movements are hampered or reduced by EP’s inhibitory action on the thalamus, this activity might simultaneously also inhibit the APP to such an extent that it results in a diminished excitatory drive to IO. A reduced or changed olivary activity could reduce or influence the learning potential of the cerebellum. This raises the interesting possibility that inhibition of movements by the basal ganglia also effectively reduces cerebellar learning potential. This idea seems testable in both animal as well as human settings.

In line with this are recently evolved ideas that olivary climbing fibers may be involved in reward signaling (Kostadinov et al., 2019). Hence, a robust basal ganglia connection with the APP could also serve to allow or curb transmission of signals that monitor reward expectation to the IO. As such, basal ganglia mechanisms involved in reinforcement learning may find its way to the olivocerebellar circuitry (Kostadinov and Hausser, 2022).

## Data availability statement

The raw data supporting the conclusions of this article will be made available by the authors, without undue reservation.

## Ethics statement

The animal study was reviewed and approved by the Ethics Committee in Neuroscience at the INT, Marseille (nr. 02 167.01) and the Institutional Animal Welfare Committee of Erasmus MC (15-273-146 and 15-273-147).

## Author contributions

TR conceptualized, designed, and carried out the experiments together with PC and XW. ES-G provided histology and part of the analysis. ZG provided funds and materials. TR wrote the manuscript and designed the figures. All authors provided edits and approved the submitted version of the manuscript.

## Footnotes

1
https://mouse.brain-map.org/

2
https://connectivity.brain-map.org

3
https://connectivity.brain-map.org/projection/experiment/305024724

4
https://connectivity.brain-map.org/projection/experiment/539498984
