# Supplementary material for: A disynaptic basal ganglia connection to the inferior olive: potential for basal ganglia influence on cerebellar learning
Source: Front Syst Neurosci. 2023 May 5;17:1176126. doi: 10.3389/fnsys.2023.1176126 (PMC10196041; doi:10.3389/fnsys.2023.1176126)
Supplement: Supplementary file 1 [file Data_Sheet_1.docx]

**Supplementary material**

**Figures**

**
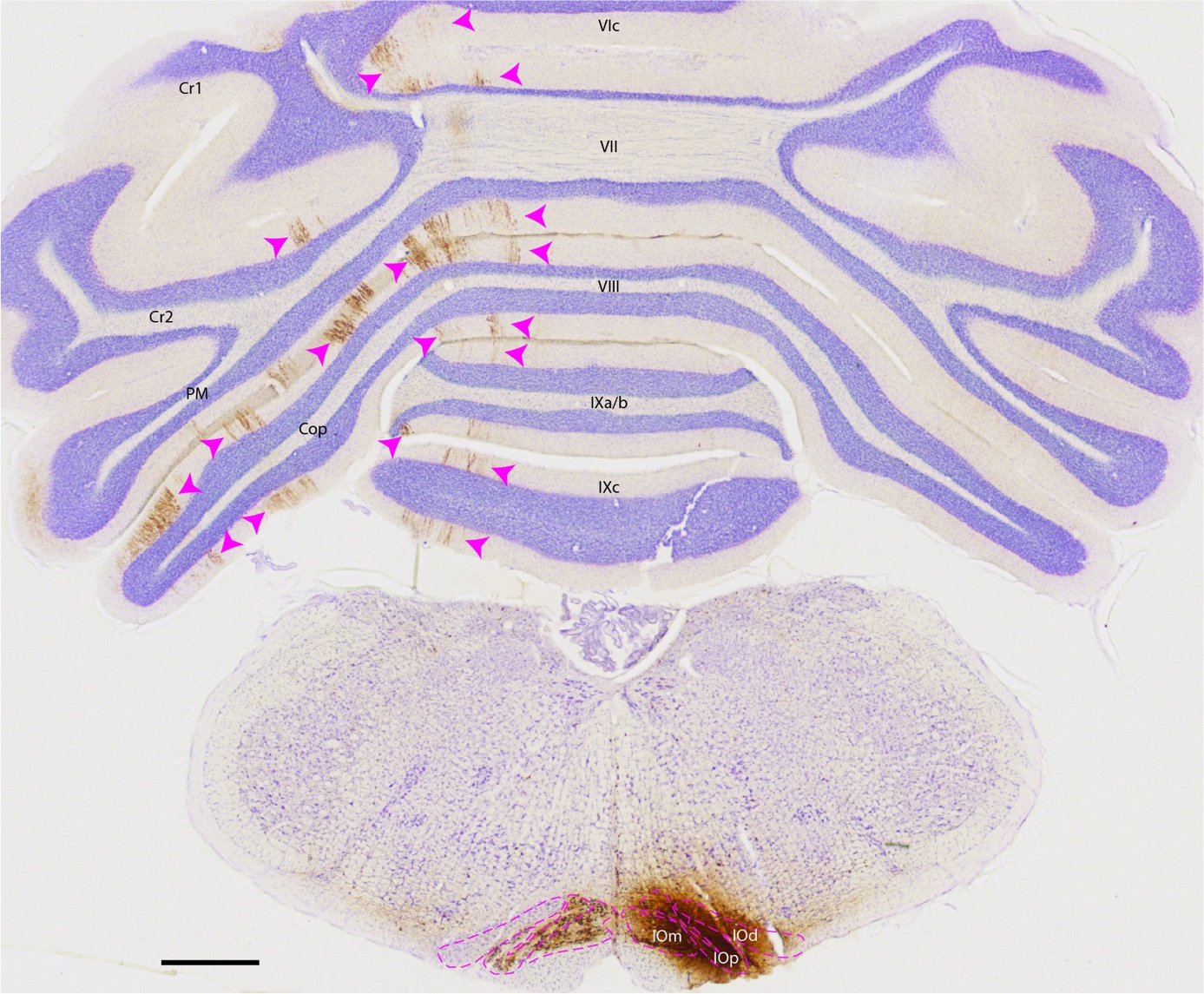
**

**Figure S1**. CTb/RABV injection in the IO of the rat results in climbing fiber labeling in molecular layer of the contralateral cerebellar cortex. Climbing fibers are indicated by magenta arrowheads. Abbreviations: VIc, VII, VIII, IXa,b, IXc: vermal lobules of the rat cerebellum; Cr1, crus 1; Cop, copula pyramidis; Cr2, crus 2; IOd, dorsal accessory nucleus of the inferior olive (IO); IOm, medial accessory nucleus of the IO; IOp, principal nucleus of the IO; PM, paramedian lobule. Scale bar equals 1 mm.

**
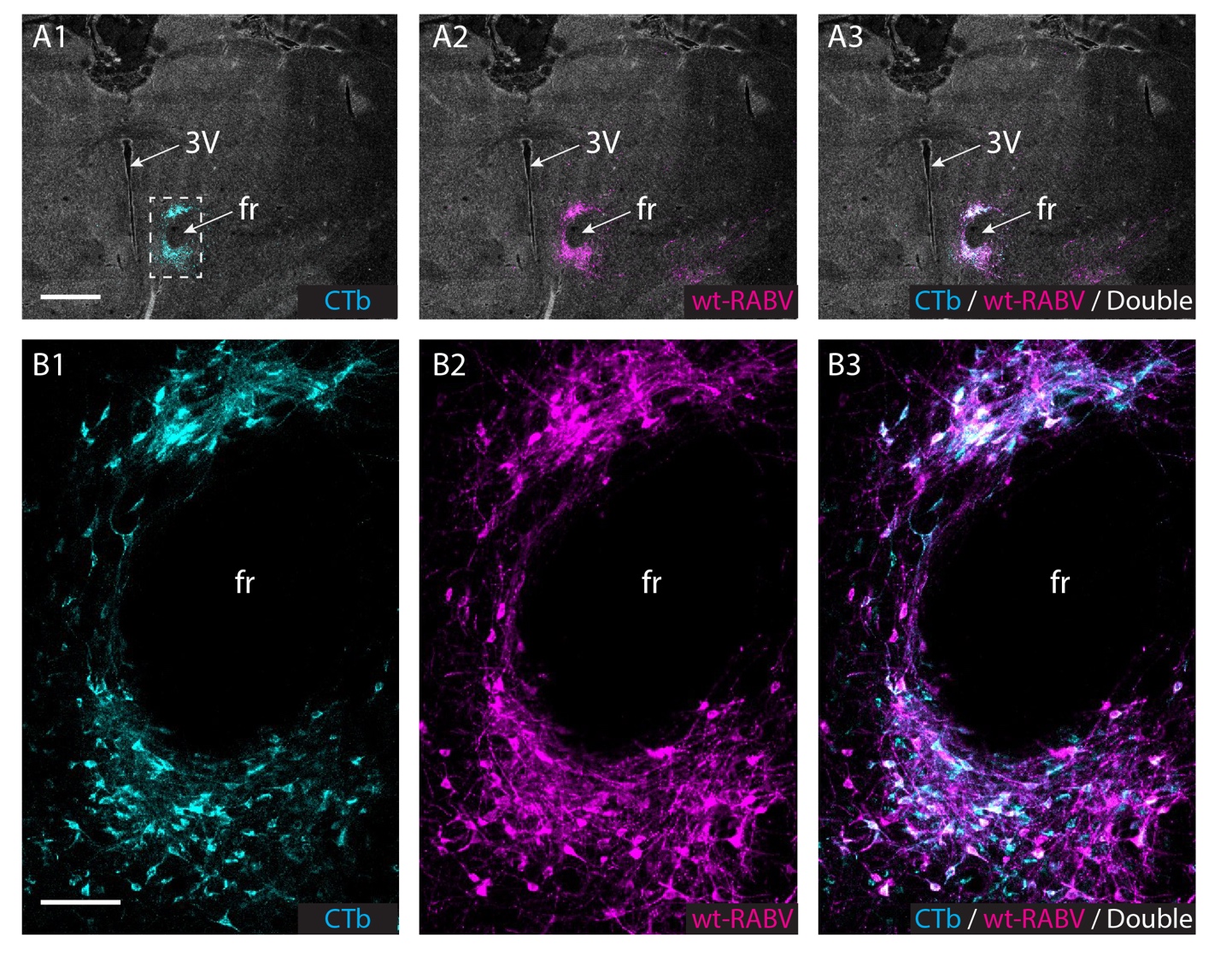
**

**Figure S2**. CTb-labeled cells around the fr can also be infected by RABV, 48-50 hours after a joint injection of CTb and RABV in the inferior olive of the rat. Overview (**A1-A3**) and detail (**B1-B3**) of boxed area in A1 of a section at the mesodiencephalic level showing CTb labeled neurons around the fr in cyan (**A1, B1**). At the same location RABV-infected neurons are located (magenta: **A2, A3**). Many neurons contain both labels (white: **A3, B3**). 3V, 3^rd^ ventricle; fr, fasciculus retroflexus. Scale bar equals 500 µm in (**A**) and 100 µm in (**B**).

**
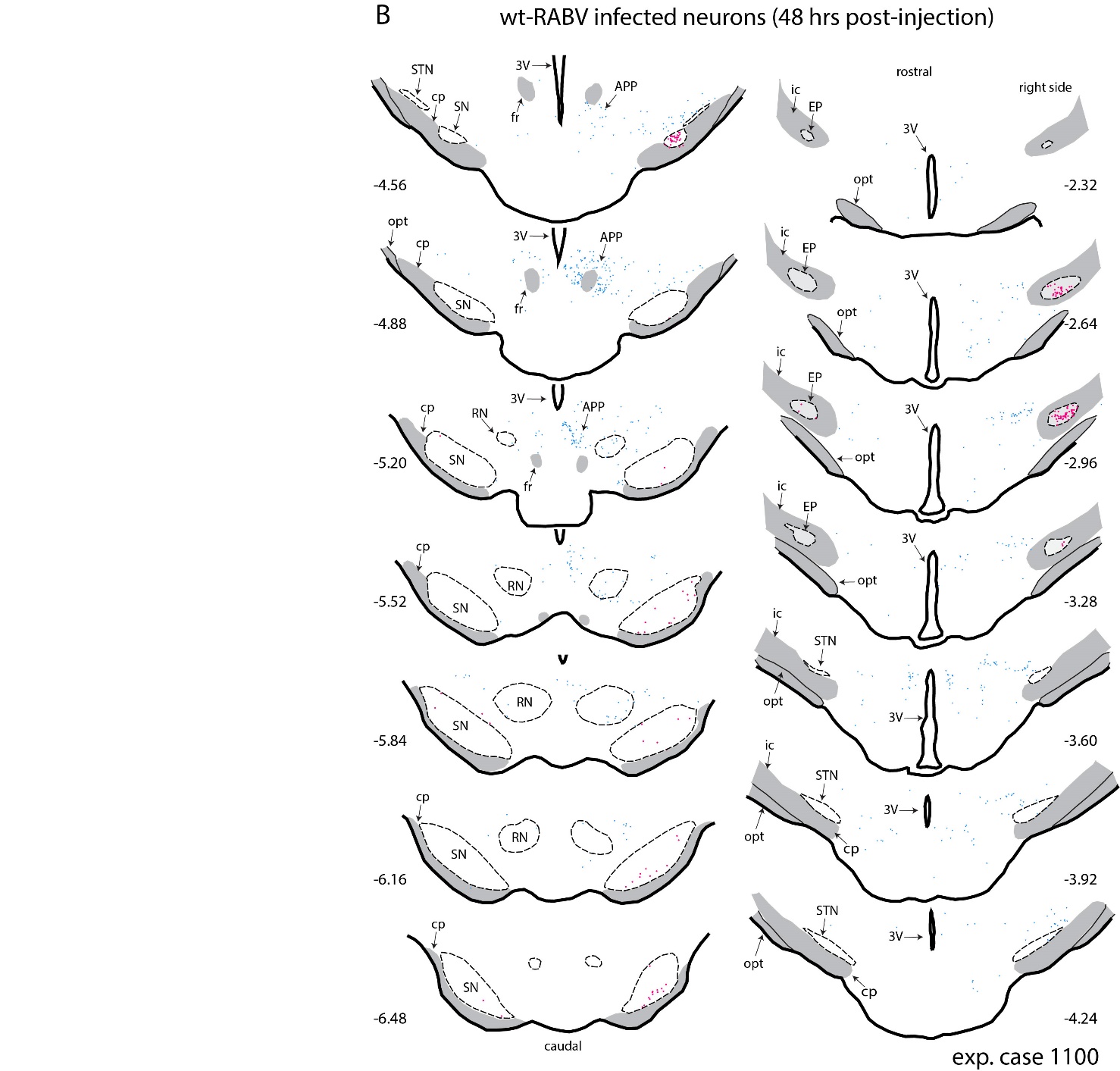
**

**
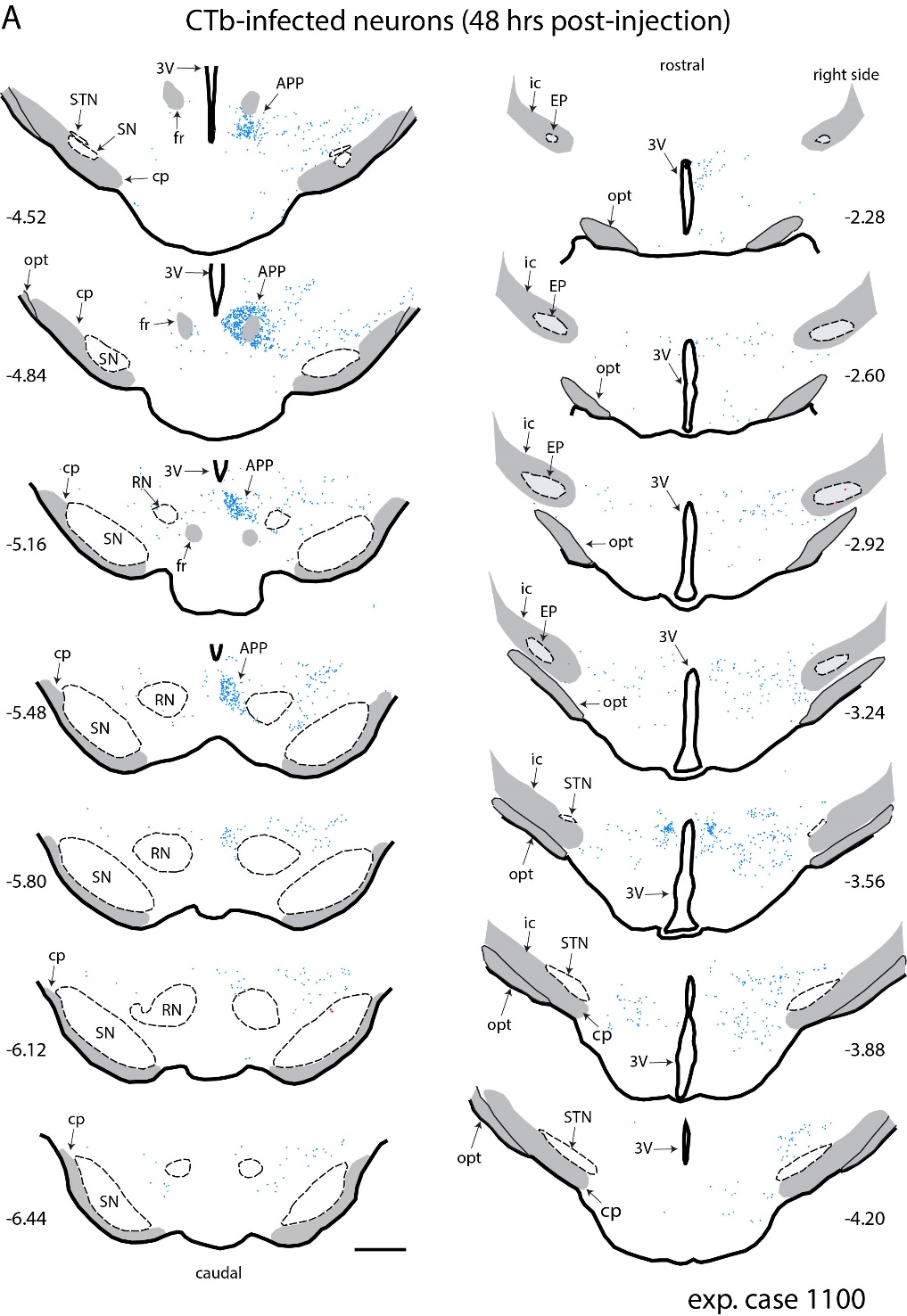
**

**Figure S3.** Plots of CTb-labeled (**A**) and RABV-infected (**B**) neurons in the mesodiencephalic region in experimental case 1100 at a post-injection time of 48 hrs. Only every second plot (i.e. plots are at 320 µm intervals) that was incorporated in the 3D-reconstruction of Fig. 2A is shown. Every blue dot represents one labeled neuron. Labeled neurons in the EP or SN are indicated with red dots. Note abundant red labeled dots in the rostral tip of the SN and the EP in (B). Abbreviations: 3V, 3^rd^ ventricle; APP, area parafascicularis prerubralis; cp, cerebral peduncle; EP, entopeduncular nucleus; fr, fasciculus retroflexus; ic, internal capsule; opt, optic tract; SN, substantia nigra; STN, subthalamic nucleus. Approximate position of sections with the respect to bregma is indicated in lower left hand corner (Paxinos and Watson, 2007). Scale bar equals 1 mm.

**
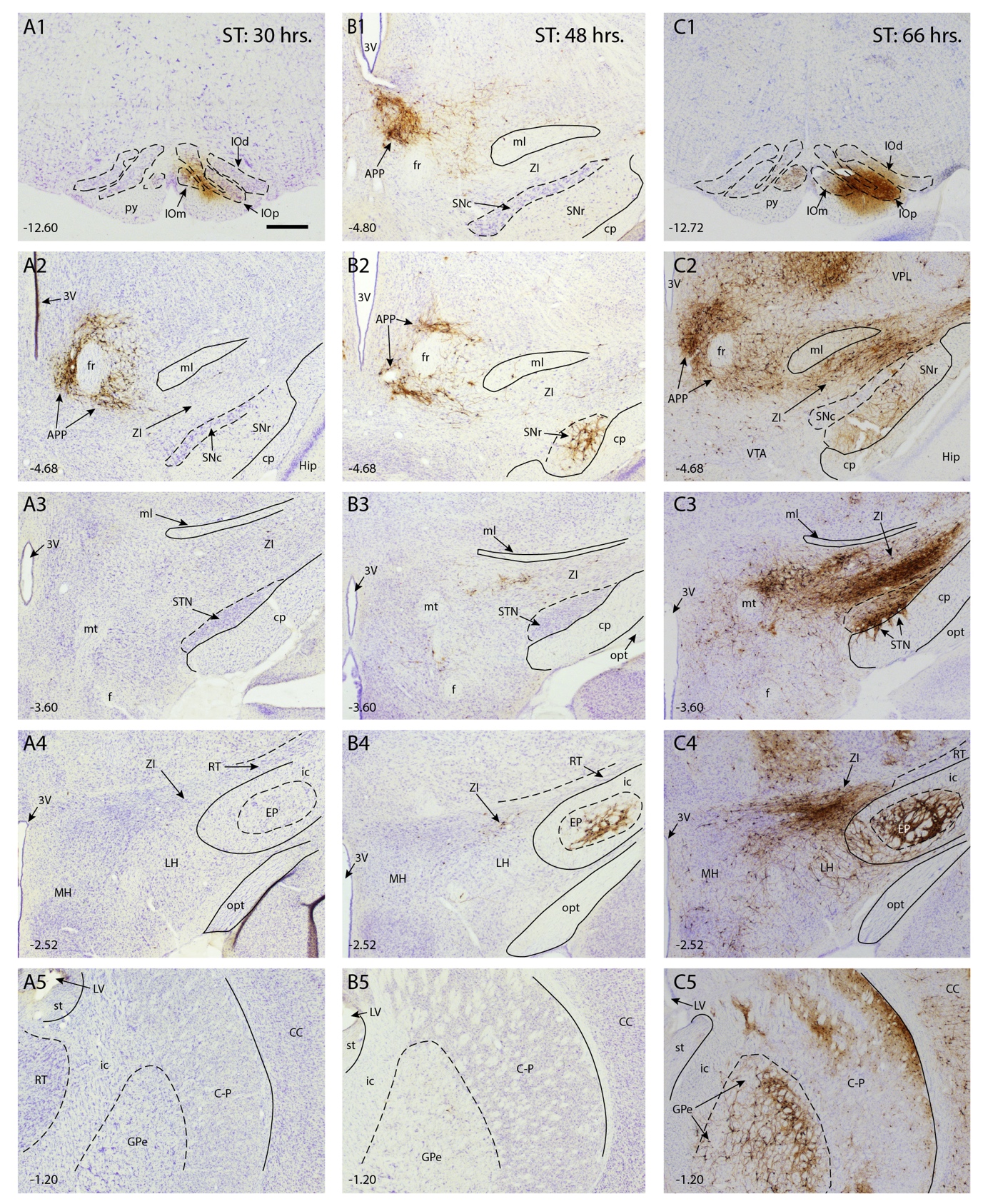
**

**Figure S4**. Progression of wt-RABV infection at 30- (**A1-5**), 48- (**B1-5**) and 66-hours (**C1-5**) post-injection in the IO of the rat. (**A1-5)** shows the injection site (with CTb immunostaining: **A1**), RABV-infection within the APP (**A2**), but no labeling within the STN (**A3**), EP (**A4**) or other parts of the basal ganglia (GPe and C-P: **A5**). (**B1-5)** shows that apart from labeling in the APP (**B1,2**) additional labeling in the rostral-most part of the SN (**B2**) and EP (**B4**) can be observed, both representing a first retrograde transneuronal step of the RABV. No infection of neurons in the STN, GPe or C-P was noted (**B5**). (**C1-C5)** shows the CTb injection site (**C1**) resulting in heavy RABV infection of the APP, ZI, SN and other mesodiencephalic regions (**C2**) as well as heavy infection of the EP and LH (**C4**). At this time (66 hours post-injection) also the STN (**C3**), GPe and C-P display many RABV-infected neurons (**C5**), in accordance with a second retrograde transneuronal step of the RABV infection. Abbreviations: 3V, 3^rd^ ventricle; APP, area parafascicularis prerubralis; CC, cerebral cortex; cp, cerebral peduncle; C-P, caudate-putamen; EP, entopeduncular nucleus; fr, fasciculus retroflexus; GPe, globus pallidus external part; Hip, hippocampus; ic, internal capsule; IOd, dorsal accessory nucleus of the inferior olive (IO); IOm, medial accessory nucleus of the IO; IOp, principal nucleus of the IO; LH, lateral hypothalamus; LV, lateral ventricle; MH, medial hypothalamus; ml, medial lemniscus; opt, optic tract; py, pyramidal tract; RT, reticular thalamic nucleus; SNc, substantia nigra pars compacta; SNr, substantia nigra part reticulata; st, stria terminalis; STN, subthalamic nucleus; VPL, ventral posterolateral thalamic nucleus; VTA, ventral tegmental area; ZI, zona incerta. Approximate position of sections with the respect to bregma is indicated in lower left hand corner (Paxinos and Watson, 2007). Scale bar equals 500 µm.

**
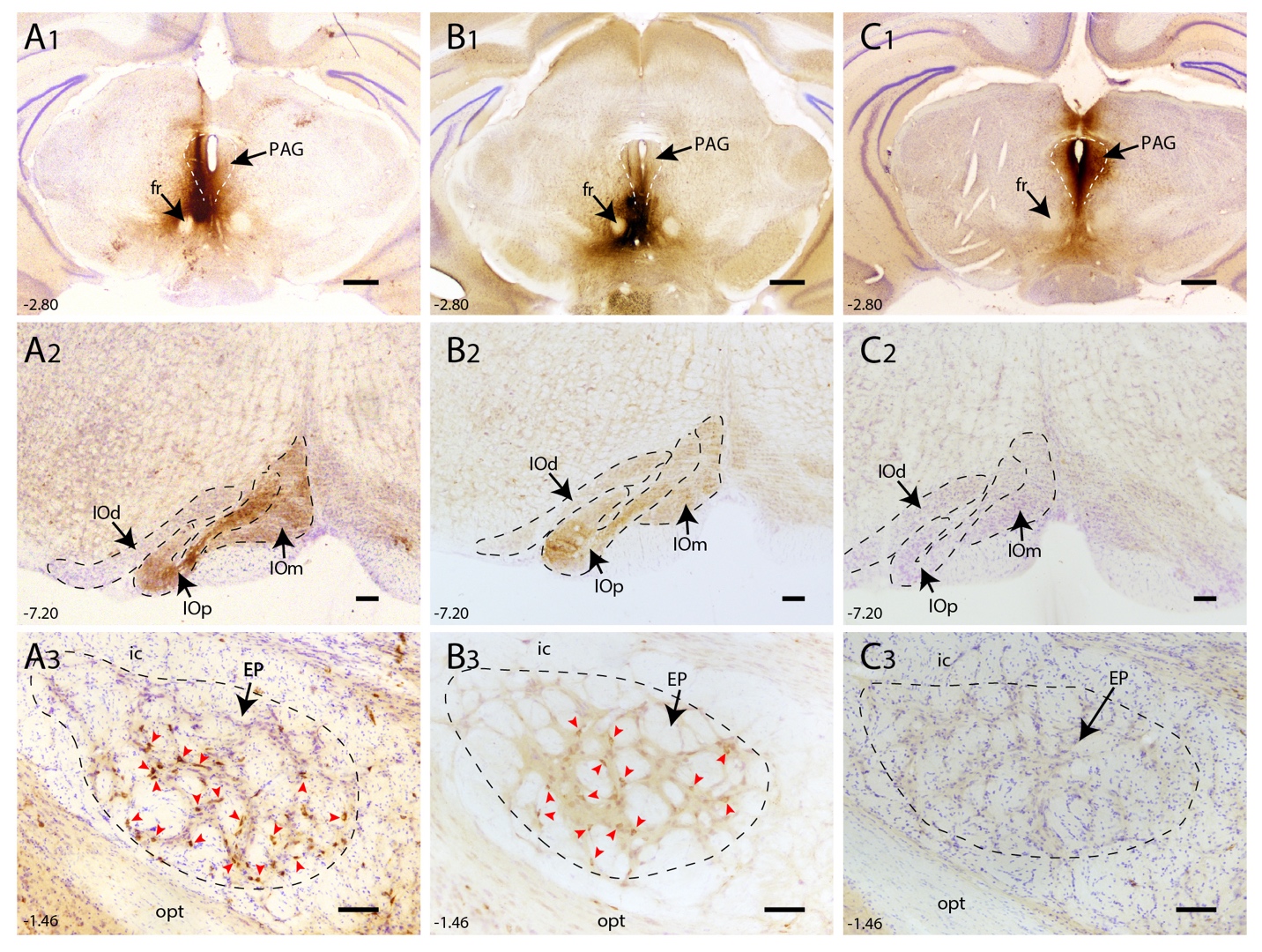
**

**Figure S5.** CTb injections in the left APP of the mouse result in anterograde labeling in the IO and retrograde labeling in the EP. (**A1-3**) and (**B1-B3**) show two examples of CTb injections centered on the APP (**A1, B1**), both resulting in anterograde terminal labeling in the ipsilateral IOp and IOm (**A2, B2**) and retrogradely labeled neurons in the ipsilateral EP (red arrowheads). In contrast when the CTb injection was made just medial to the EP (**C1**), no olivary labeling (**C2**) or EP labeling (**C3**) could be discerned. Abbreviations: EP, entopeduncular nucleus; fr, fasciculus retroflexus; ic, internal capsule; IOm, medial accessory nucleus of the IO; IOp, principal nucleus of the IO; opt, optic tract. Approximate position of sections with the respect to bregma is indicated in lower left hand corner (Paxinos and Franklin, 2004). Scale bar equals 500 µm in A1, B1 and C1 and 100 µm in the other panels.


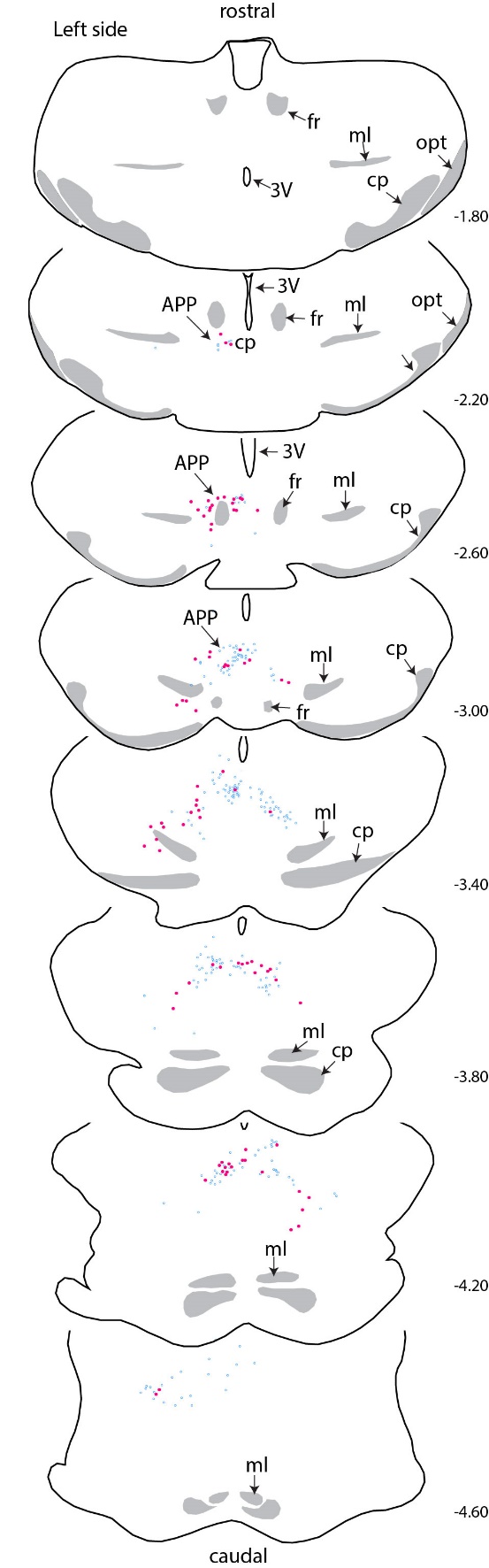


**Figure S6.** Sequential coronal plots of starter cells in the mesodiencephalic region in experimental case 12174-05. Only every second plot (i.e. plots are at 400 µm intervals) that was incorporated in the 3D-reconstruction of Fig. 5C is shown. Every dot represents one labeled neuron. Magenta dots signify cre-labeled neurons infected with RABV, representing starter cells. Cyan dots signify cre-labeled neurons only. Note that most starter cells are found in the APP region. Abbreviations: 3V, 3^rd^ ventricle; APP, area parafascicularis prerubralis; cp, cerebral peduncle; fr, fasciculus retroflexus; ml, medial lemniscus; opt, optic tract. Approximate position of sections with the respect to bregma is indicated in lower left hand corner (Paxinos and Franklin, 2004). Scale bar equals 1 mm.


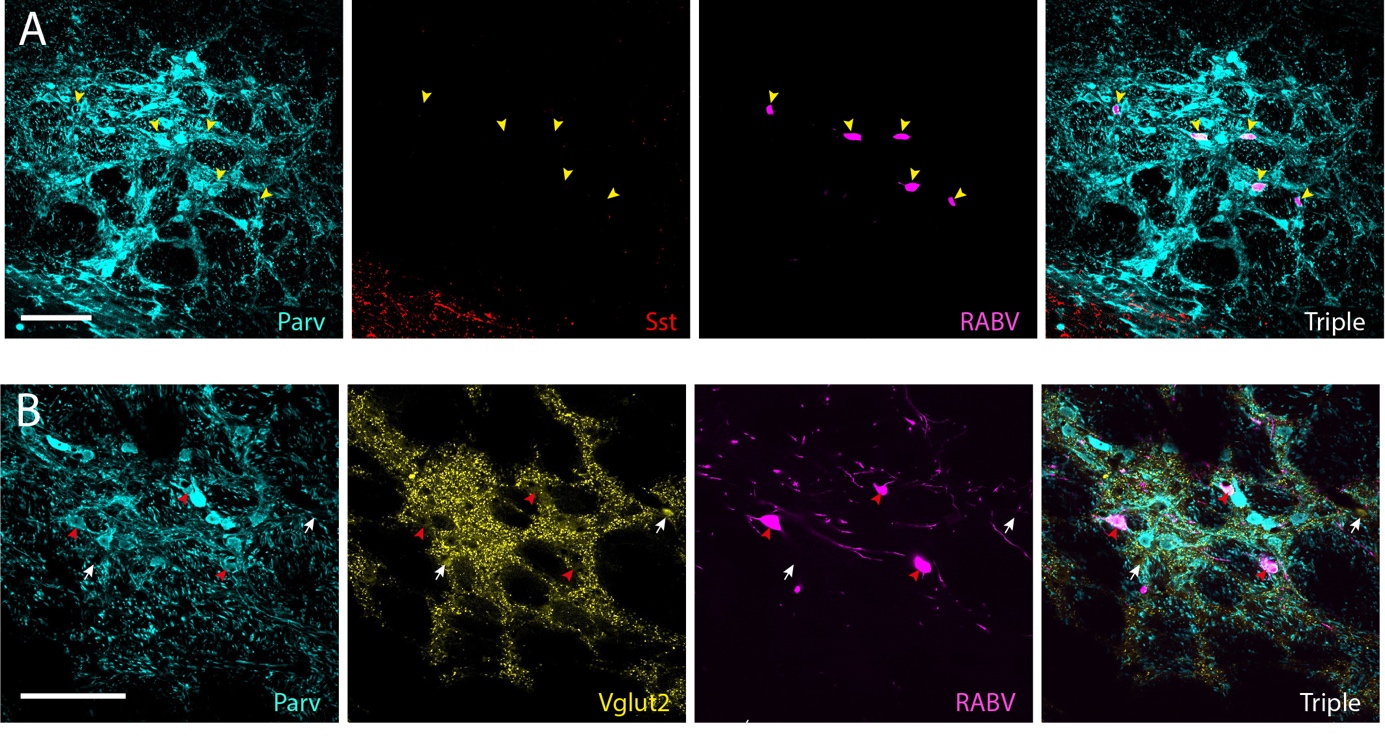


**Figure S7.** Triple-labeling of RABV, Parv, and Sst (**A**) or Vglut2 (**B**) in the mouse EP. (**A**) The position of RABV-labeled neurons are indicated in all panels by yellow arrow heads. Note that no Sst-labeled somata can be observed within this section. All RABV profiles are Parv+. (**B**) For this section RABV labeled neurons are indicated with red arrow heads in all panels. All RABV neurons are Parv+ and Vglut2-. Two potentially Vglut2+ somata are indicated by white arrows in all panels. Scale bar equals 100 µm.


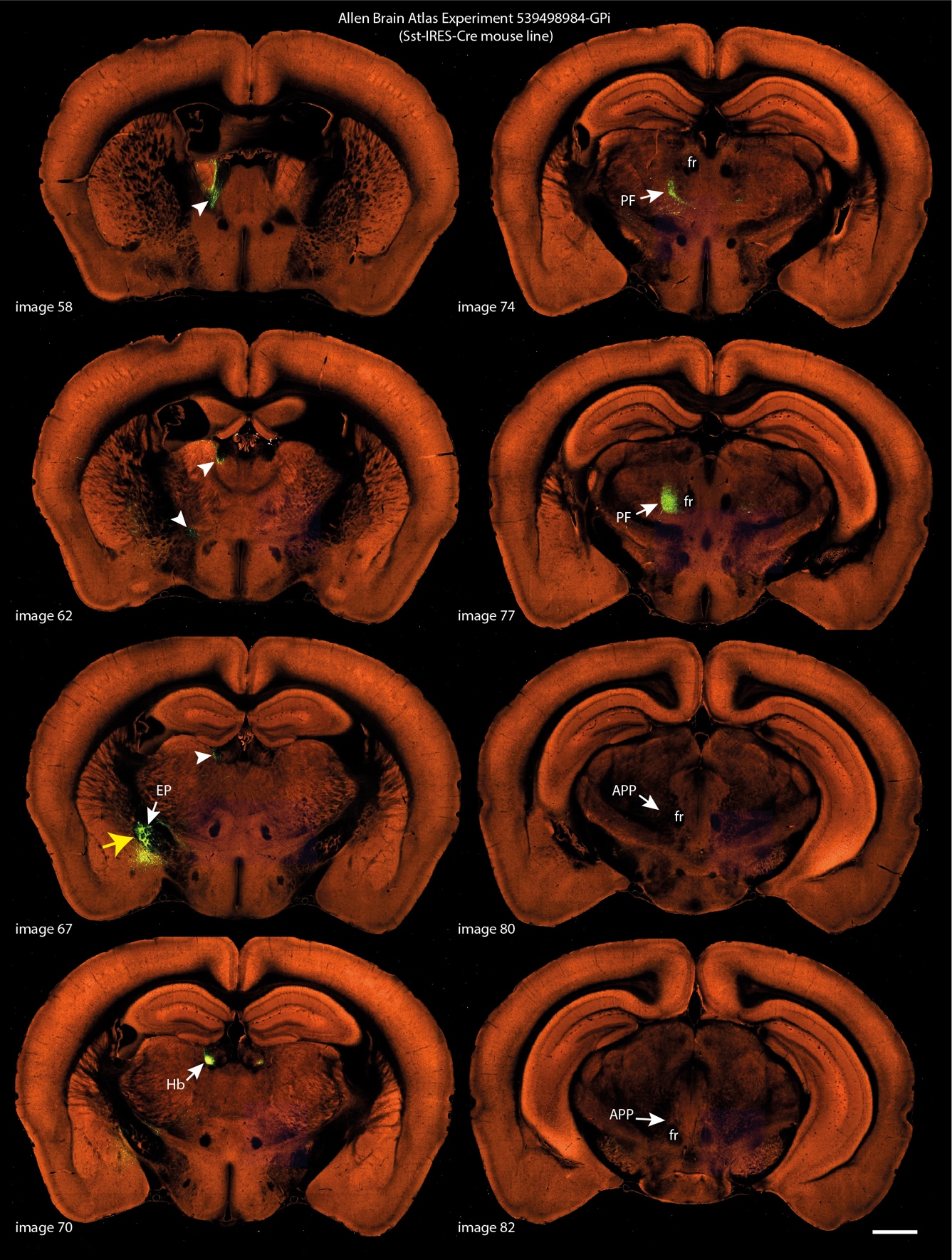


**Figure S8**. Sst-positive neurons do not project to the APP. Sequential images from the Mouse Connectivity Atlas, experiment 539498984-GPi from the Allen Brain Atlas (<https://connectivity.brain-map.org/projection/experiment/539498984>; Allen-Institute-for-Brain-Science, 2004). The injection (yellow arrow) involving the EP was made in the Sst-IRES-Cre mouse line. Labeled fibers to the habenula can be traced to follow the thalamic stria medullaris (arrow heads). Additional terminal tracing can be observed in the parafascicular thalamic nucleus. However, no labeling is observed in the APP. Image numbers correspond to numbers on the Allen Brain website. Abbreviations: APP, area parafascicularis prerubralis; EP, entopeduncular nucleus; fr, fasciculus retroflexus; Hb, habenula; PF, parafascicular thalamic nucleus. Scale bar equals 1 mm.
